# A study for precision diagnosing and treatment strategies in difficult-to-treat AIDS cases and HIV-infected patients with highly fatal or highly disabling opportunistic infections: Study protocol for antiretroviral therapy timing in AIDS patients with toxoplasma encephalitis

**DOI:** 10.1097/MD.0000000000021141

**Published:** 2020-07-17

**Authors:** Yan-Ming Zeng, Yao Li, Xiao-Qing He, Yin-Qiu Huang, Min Liu, Jing Yuan, Yan Bai, Yan-Qiu Lu, Huan Li, Yao-Kai Chen

**Affiliations:** Division of Infectious Diseases, Chongqing Public Health Medical Center, Chongqing, China.

**Keywords:** acquired immunodeficiency syndrome, antiretroviral therapy, timing, toxoplasma encephalitis

## Abstract

**Background:**

Toxoplasma encephalitis (TE) is one of the main opportunistic infections in acquired immunodeficiency syndrome (AIDS) patients, and represents a social burden due to its high prevalence and morbidity. Concomitant antiretroviral therapy (ART), together with effective anti- toxoplasma combination therapy, is an effective strategy to treat AIDS-associated TE (AIDS/TE) patients. However, the timing for the initiation of ART after diagnosis of TE remains controversial. We therefore designed the present study to determine the optimal timing for ART initiation in AIDS/TE patients.

**Methods/Design:**

This trial is a 17-center, randomized, prospective clinical study with 2 parallel arms. A total of 200 participants will be randomized at a 1:1 ratio into the 2 arms: the early ART initiation (≤14 days after TE diagnosis) arm and the deferred ART (>14 days after TE diagnosis) arm. The primary outcome will be the difference of mortality between the 2 arms at 48 weeks. The secondary outcomes will be the differences between the 2 arms in the changes of CD4+ counts from baseline to week 48, the rate of virologic suppression (HIV ribonucleic acid <50 copies/mL) from baseline to week 48, the incidence of TE-associated immune reconstitution inflammatory syndrome during the study period, and the incidence of adverse effects during the study period.

**Discussion:**

This present trial aims to evaluate the optimal timing for ART initiation in AIDS/TE patients, and will provide strong evidence for AIDS/TE treatment should it be successful.

**Trial registration:**

This trial was registered as one of the 12 trials under the name of a general project at the chictr.gov (http://www.chictr.org.cn/showproj.aspx?proj=35362) on February 1, 2019, and the registration number of the general project is ChiCTR1900021195.

## 1 Introduction

Although the overall mortality of HIV infections is declining,^[1]^ the number of new HIV infections per year, and the number of deaths related to acquired immunodeficiency syndrome (AIDS)-associated opportunistic infections (OIs) are still significantly large, and thus, HIV/AIDS remains one of the world's largest public health challenges.^[2]^

Toxoplasma infection often affects the central nervous system, resulting in toxoplasma encephalitis (TE),^[3]^ a life-threatening disease and one of the commonest OIs of the central nervous system in AIDS patients with CD4+ counts <200 cells/μl. Before the advent of antiretroviral therapy (ART), the 12-month incidence of TE was approximately 33% in patients with advanced immunosuppression who were seropositive for *Toxoplasma gondii*, and not receiving prophylaxis drugs against the disease.^[4]^ In current times, the widespread application of ART and trimethoprim-sulfamethoxazole prophylaxis has greatly reduced the prevalence of toxoplasma infection, however, the number of TE cases and TE-associated deaths is still substantial, especially in resource-limited regions where the coverage of ART is not high.

The decision concerning when to initiate ART in AIDS patients with TE (AIDS/TE), is among the most vexing issues in the domain of AIDS therapy.^[5]^ It is currently acknowledged that modern ART can reduce HIV viral load effectively and rapidly, resulting in immune reconstitution, and that early initiation of ART improves prognosis in AIDS patients even more greatly.^[6]^

Despite the success of ART in treating AIDS patients, initiating ART too early should be cautious in patients with OIs including those with TE, due to the concerns of immune reconstitution inflammatory syndrome (IRIS), drug-drug interactions and drug toxicities.^[7]^ The development of IRIS may have significant negative implications for TE patients, for example, as the emergence of IRIS may result in rehospitalization, changes to therapy, or even deaths.^[8]^ There are very limited data in the literature at present regarding the timing of ART when TE is present in ART-naive patients. We therefore designed the present study to determine the optimal timing for ART initiation in AIDS/TE patients.

## 2 Methods/design

### 2.1 Research objective

This study aims to investigate the most optimal time for ART initiation in AIDS/TE patients.

### 2.2 Study design

This trial is designed as an open-labelled, multicenter, prospective, randomized, and controlled clinical study. Two hundred patients will be recruited in the following 17 hospitals in mainland China: Chongqing Public Health Medical Center, Beijing Youan Hospital of Capital Medical University, the Forth Affiliated Hospital of Harbin Medical University, the Second People's Hospital of Tianjin, the First Hospital of Changsha, the Eighth People's Hospital of Guangzhou, Liuzhou General Hospital, the Third People's Hospital of Guilin, the Third People's Hospital of Shenzhen, Guiyang Public Health Clinical Center, Public Health Clinical Center of Chengdu, the Third People's Hospital of Kunming, Yunnan Provincial Infectious Disease Hospital, the Fourth People's Hospital of Nanning, Longtan Hospital of Guangxi Zhuang Autonomous Region, the First Affiliated Hospital of Zhejiang University, and Xixi Hospital of Hangzhou. All enrolled patients will be randomized into 2 arms: the early ART initiation (≤14 days after diagnosis) arm, and the deferred ART (>14 days after diagnosis) arm. This study will recruit a total of 200 participants, with 100 subjects in each arm.

Each individual will be assessed in a 48-week follow-up period at day 0, week 4, week 8, week 12, week 24, week 36, and week 48. Blood and urine samples will be collected for laboratory testing, including hematological analysis, liver and kidney function test, electrolyte analysis, urinalysis, urine pregnancy test, anti-toxoplasma immunoglobin G / immunoglobin M antibody test, lymphocyte subset test, HIV-1 ribonucleic acid (RNA) quantification test, electrocardiogram, and head magnetic resonance imaging / computed tomography scan. Table 1 summarizes all the tests to be conducted during the study period.

**Table 1:**
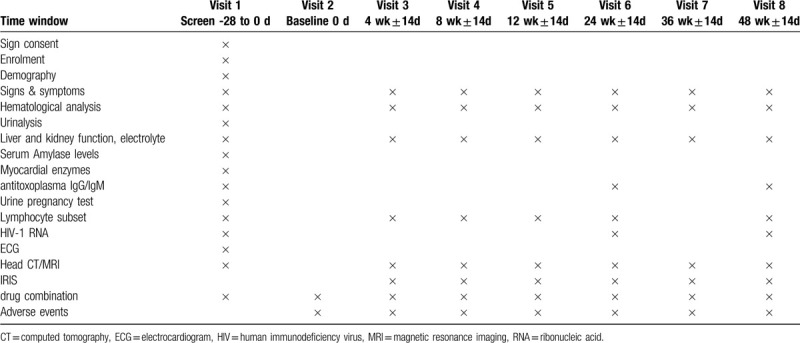
Study time points of enrollment, intervention, and assessment.

### 2.3 Participants

#### 2.3.1 Diagnostic criteria

The diagnostic criteria of HIV infection and AIDS for this trial are consistent with the Department of Health and Human Services Guidelines for the Prevention and Treatment of Opportunistic Infections in HIV-Infected Adults and Adolescents^[4]^ and the 2018 Chinese Guidelines for Diagnosis and Treatment of HIV/AIDS.^[9]^

The diagnosis of TE needs to satisfy the following criteria:

(1) Clinical presentations such as headache, confusion, motor weakness or fever;
(2) Computed tomography or magnetic resonance imaging scans show cerebral ring-enhancing lesions, especially in the basal ganglia;
(3) Positivity for antitoxoplasma immunoglobin G/ immunoglobin M antibody test, toxoplasma antigen test, toxoplasma DNA detection by polymerase chain reaction, or t*oxoplasma gondii* staining;
(4) Effective antitoxoplasma treatment.

TE-associated IRIS (TE-IRIS) is diagnosed according to the following criteria:

(1) Anti-toxoplasma drug treatment is effective, and symptoms and signs of TE have improved or disappeared prior to ART initiation;
(2) ART is effective, resulting in a decrease in plasma HIV RNA level >1 log_10_ copies/ml or increase in CD4+ count of ≥25 cells/μl after ART initiation;
(3) Patients show worsening clinical symptoms or radiological signs of TE after ART initiation and the worsening cannot be explained by drug toxicity or newly acquired disease.

#### 2.3.2 Inclusion criteria

(1) Adult (defined as ≥18 years of age);
(2) Confirmed HIV infection;
(3) Diagnosed with TE;
(4) ART-native;
(5) Willingness to give an informed consent.

#### 2.3.3 Exclusion criteria

(1) Allergic or intolerant to ART drugs;
(2) Hemoglobin <60 g/L, white blood cell count <1.0 × 10^9^/L, neutrophil count <0.5 × 10^9^/L, platelet count <50 × 10^9^/L, blood amylase >2 × UNL, serum creatinine >1.5 × UNL, aspartate aminotransferase /alanine aminotransferase /alkaline phosphatase >5 times of UNL, total bilirubin >2 × UNL, serum creatine phosphokinase >2 × UNL;
(3) Pregnancy or breast-feeding;
(4) Severe cardiovascular disease, liver disease, kidney disease, lung disease, or other diseases that may reduce patient compliance;
(5) Severe mental illness;
(6) Patients with intravenous recreational drug use;
(7) Non-Chinese nationality.

#### 2.3.4 Recruitment

**Figure 1:**
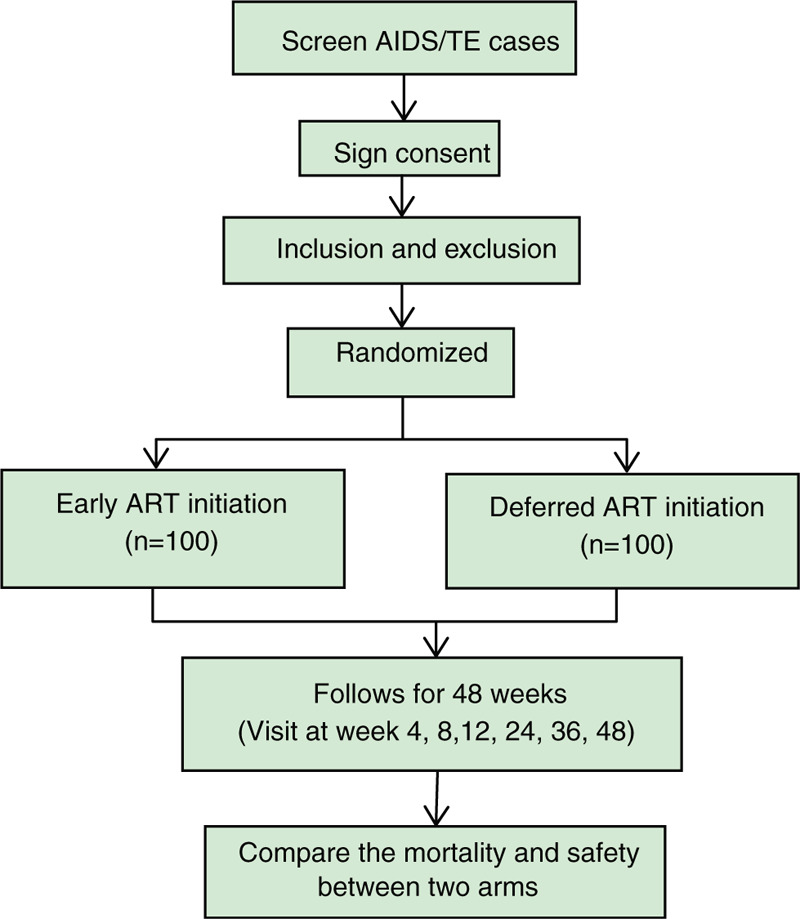
Flow chart of study design.

Co-principal investigators (Co-PIs) from each center will supervise the enrollment procedure and ensure the proposed sample size is attained. Physicians will evaluate subjects in the inpatient wards. Individuals will be selected according to inclusion and exclusion criteria specified by this protocol. The trial flow diagram is illustrated in Figure 1.

#### 2.3.5 Randomization

Patients will participate voluntarily and will sign an informed consent form before screening. All eligible patients will be randomly assigned to one of the 2 arms at a 1:1 ratio by Medical Research Platform (http://www.51yyt.org/FrontPage/Index.aspx).

#### 2.3.6 Intervention

Patients in both arms will receive antitoxoplasma treatment at least 6 weeks with trimethoprim-sulfamethoxazole (1.44 g, PO, tid) and azithromycin (0.5 g/d, ivgtt, qd) due to inaccessibility of pyrimethamine and sulfadiazine in China. For some patients who have no obvious improvement, the regimen will be replaced or the course will be extended. Our study will not limit the choice of ART regimen selected for each participant.

#### 2.3.7 Endpoints

(1) Completion of the 48-week follow-up as per the study protocol;
(2) Discontinuation of ART during the study period;
(3) Death.

#### 2.3.8 Outcomes

##### 2.3.8.1 Primary outcome

The difference in overall mortality between the 2 study arms at week 48.

##### 2.3.8.2 Secondary outcomes

(1) The difference in the change of CD4+ cell counts between the 2 study arms from baseline to week 48;
(2) The difference in the rate of virologic suppression (HIV RNA <50 copies/mL) between the 2 study arms at week 48;
(3) The difference in the incidence of TE-IRIS between the 2 study arms during the study period;
(4) The difference in the incidence of Adverse events (AEs) between the 2 study arms during the study period.

#### 2.3.9 Sample size

The sample size will be 100 per arm based on a power of 80% with a level of confidence of 95%. We expected the dropout rate will be 15%.

### 2.4 Statistical analysis

All study variables will be presented, and clinical and demographic characteristics will be summarized and reported using descriptive statistics. Categorical variables will be summarized as numbers and percentages, and will be compared using the chi-squared test or the Fisher exact test. Continuous variables will be summarized as medians and interquartile ranges or as means and standard deviations, and will be compared using the Mann-Whitney *U* test or Student *t* test, as appropriate. The time-to-event method, with Cox proportional-hazards models, will be used to compare the mortality at week 48. All the analyses will be carried out using the SPSS program (IBM Corp, Armonk, NY). The level for statistical significance will be set at 5%.

#### 2.4.1 Data collection

The collection of clinical information from patients will begin at baseline, and will continue with follow-up as established and defined in the study. All collected data will be recorded on case report forms (CRFs) and in the database via the Medical Research Platform. The monitor will review the CRFs and ensure that information on the CRFs is in agreement with those in the original medical records. After the study, the suitability of the database for analysis will be considered, and the database will then be locked by the PI, statistical analysts and data managers.

#### 2.4.2 Patient safety

AEs will be continuously monitored for 48 weeks during the study period, and the incidence of AEs will be evaluated at each follow-up visit. All specific AEs will be documented in the individual participant CRF, with appropriate medical terminology, including occurrence time, duration, severity, and will be classified as mild, moderate, or severe. Any prolonged hospitalization, persistent or significant disability or incapacity, or unexpected medical occurrence that results in death will be reported as a severe AE, which will need to be reported to the PI and the ethics committee within 24 hours.

#### 2.4.3 Quality control

Throughout the study the PI will monitor the quality of the trial. To maintain data quality and integrity, and to ensure adherence to this protocol:

(1) all investigators will be trained rigorously based on a standardized operation practice manual;
(2) Startup meetings will be held for all sites;
(3) Monitoring visits will take place before, during, and at the completion of the study.

## 3 Discussion

TE is an HIV-indicative event in 35% of patients and an AIDS-defining event in 75% of cases,^[10]^ and is associated with significant mortality up to 29.9%.^[11]^ In the absence of treatment, TE may cause epilepsy, coma, and death in AIDS patients. For AIDS/TE patients, ART plays a vital role in saving life and preventing disability. However, there are currently no evidence-based guidelines recommending the optimal timing for ART initiation in AIDS/TE patients. This dearth of clinically based recommendations has resulted in divided expert opinion, and inconsistencies among current treatment guidelines. We therefore wish to conduct a randomized clinical trial to compare authentic clinical data of therapeutic efficacy and safety between early ART initiation and deferred ART initiation in AIDS/TE patients.

Previous studies have made some investigation of this issue. However, the number of participants with AIDS/TE in those studies is pretty small and it is not realistic to draw a substantial conclusion from previous studies. Schafer et al, for example, investigated the timing of ART initiation in AIDS patients with OIs, including 11 patients with TE, and they found that there were no significant differences in efficacy and safety between the immediate ART initiation arm (within 7 days of TE treatment) and the deferred initiation arm (after the completion of TE treatment, ranging from 28 days to 6 weeks).^[7]^ Zolopa et al carried out a multicenter randomized trial which enrolled 282 patients with OIs, finding that compared with deferred ART initiation (after the completion of the OIs treatment), the early ART arm (within 14 days of OIs treatment) had fewer AIDS progression/deaths and a longer time to AIDS progression/death.^[12]^ However, only 13 AIDS/TE patients were included in this study.

So far, we have not found any randomized cohort studies concerning the optimal timing for ART initiation in AIDS/TE patients, indicating that conducting randomized multicenter prospective trials in this population is urgently warranted. We expect that the results of this study will be able to provide solid evidence for the clinical management of patients with AIDS/TE.

### 3.1 Trial status

This trial has been approved by the Institutional Review Boards at the 17 involved hospitals. Enrollment for this study began in March 2019, and 66 patients have been enrolled as at May 31, 2020. At present, the study is in progress. (protocol version 5, 28 August 2019). There is no publication containing the results of this study have been published or submitted.

## Acknowledgments

We appreciate the assistance of Dr. Vijay Harypursat from New Zealand for language improvement and revision of the manuscript.

## Author contributions

X-q H and Y-q H conceived the study. M L and J Y designed the study protocol. Y-m Z and Y L drafted the manuscript. Y B developed the first draft of the manuscript. Y-q L registered the study. H L and Y-k Chen sought funding and ethical approval. All authors read and approved the manuscript.
